# A Simple Mechanism for Complex Social Behavior

**DOI:** 10.1371/journal.pbio.1001039

**Published:** 2011-03-29

**Authors:** Katie Parkinson, Neil J. Buttery, Jason B. Wolf, Christopher R. L. Thompson

**Affiliations:** 1 Faculty of Life Sciences, Michael Smith Building, University of Manchester, Manchester, United Kingdom; 2 Department of Biology and Biochemistry, University of Bath, Bath, United Kingdom; University of Lausanne, Switzerland

## Abstract

The evolution of cooperation is a paradox because natural selection should favor exploitative individuals that avoid paying their fair share of any costs. Such conflict between the self-interests of cooperating individuals often results in the evolution of complex, opponent-specific, social strategies and counterstrategies. However, the genetic and biological mechanisms underlying complex social strategies, and therefore the evolution of cooperative behavior, are largely unknown. To address this dearth of empirical data, we combine mathematical modeling, molecular genetic, and developmental approaches to test whether variation in the production of and response to social signals is sufficient to generate the complex partner-specific social success seen in the social amoeba *Dictyostelium discoideum*. Firstly, we find that the simple model of production of and response to social signals can generate the sort of apparent complex changes in social behavior seen in this system, without the need for partner recognition. Secondly, measurements of signal production and response in a mutant with a change in a single gene that leads to a shift in social behavior provide support for this model. Finally, these simple measurements of social signaling can also explain complex patterns of variation in social behavior generated by the natural genetic diversity found in isolates collected from the wild. Our studies therefore demonstrate a novel and elegantly simple underlying mechanistic basis for natural variation in complex social strategies in *D. discoideum*. More generally, they suggest that simple rules governing interactions between individuals can be sufficient to generate a diverse array of outcomes that appear complex and unpredictable when those rules are unknown.

## Author Summary

Despite the appearance of cooperation in nature, selection should often favor exploitative individuals who perform less of any cooperative behaviors while maintaining the benefits accrued from the cooperative behavior of others. This conflict of interest among cooperating individuals can lead to the evolution of complex social strategies that depend on the identity (e.g. genotype or strategy) of the individuals with whom you interact. The social amoeba *Dictyostelium discoideum* provides a compelling model for studying such “partner specific” conflict and cooperation. Upon starvation, free-living amoebae aggregate and form a fruiting body composed of dead stalk cells and hardy spores. Different genotypes will aggregate to produce chimeric fruiting bodies, resulting in potential social conflict over who will contribute to the reproductive sporehead and who will “sacrifice” themselves to produce the dead stalk. The outcomes of competitive interactions in chimera appear complex, with social success being strongly partner specific. Here we propose a simple mechanism to explain social strategies in *D. discoideum*, based on the production of and response to stalk-inducing factors, the social signals that determine whether cells become stalk or spore. Indeed, measurements of signal production and response can predict social behavior of different strains, thus demonstrating a novel and elegantly simple underlying mechanistic basis for natural variation in complex facultative social strategies. This suggests that simple social rules can be sufficient to generate a diverse array of behavioral outcomes that appear complex and unpredictable when those rules are unknown.

## Introduction

Despite the appearance of cooperation in many social systems, natural selection will generally favor exploitative individuals that can maximize fitness by performing less of a costly cooperative act while maintaining the benefits accrued from the cooperative behavior of others. The evolution and maintenance of cooperation is therefore characterized by conflict between the self-interests of cooperating individuals. This social conflict can lead to the evolution of complex social strategies and counterstrategies that exploit the cooperative behavior of others while minimizing the costs of cooperation. The social amoeba *Dictyostelium discoideum* provides a compelling model for studying the genetic basis of such conflict and cooperation [1]–[5]. Upon starvation, up to 100,000 amoebae aggregate and differentiate to form a fruiting body composed of dead stalk cells that hold aloft a sporehead bearing hardy spores. Different genotypes will aggregate to produce a chimeric fruiting body, resulting in potential social conflict over which genotypes will “sacrifice” themselves to produce the stalk and which will contribute to the sporehead, and hence have direct reproductive fitness.

Naturally occurring *D. discoideum* isolates exhibit widespread variation in the total numbers of cells allocated to spores when developed clonally [1]. This has been termed a “fixed” strategy because it reflects inherent differences in allocation patterns among isolates. However, genotypes often show dramatic shifts in spore:stalk allocation in chimera (from that expected based on clonal allocations), which are highly variable and dependent on the precise pairing of genotypes or social partner [1],[6]. These changes in behavior have been termed “facultative” strategies as they produce a remarkable range of behaviors, with some genotypes showing self-promotion wherein they produce disproportionately more spores when in competition compared to that expected given their clonal allocation. Success can also be gained in chimera through coercion, where genotypes “force” other genotypes to produce more of the stalk at the expense of their own spore production. Such complexity within a small set of naturally co-occurring isolates is surprising, and it is intuitive to assume a complex underlying genetic basis such as an active recognition mechanism that causes a change in behavior in the presence of foreigners. Indeed, kin recognition has been demonstrated between geographically distant *D. discoideum* isolates [7],[8]. However, it is important to note that the description of apparently fixed and facultative behavior in *D. discoideum* is based on observations of the outcomes of interactions in clones and chimeras. It is therefore actually unknown whether it is based on a truly facultative underlying mechanism (i.e. an induced facultative shift in some underlying biological process in response to the social partner) or simply appears facultative at the behavioral level. For this reason, and to avoid confusion over descriptions of the outcomes of interactions versus the nature of the interactions themselves, hereafter we refer to these simply as clonal and chimeric strategies.

Understanding the mechanistic basis of social interactions, and more specifically, why behavior appears to change depending on social partner, is crucial for us to understand the evolution of social conflict and cooperation in *D. discoideum*, or any other social organism. Here we hypothesize that variation in clonal and chimeric social behavior in *D. discoideum* is modulated by a simple mechanism based on the production of and response to social signals that govern developmental differentiation in this system. To test this hypothesis, we examine social signaling in a collection of natural genetic isolates and also in a genotype in which we have disrupted social behavior through a mutation in a known gene. We integrate measurements of signal production and response in these genotypes with a mathematical model to examine whether we can explain the apparently complex partner-specific social behavior observed in these natural and lab-generated genotypes.

## Results

### A Model of Social Signaling in *D. discoideum*

Although social success in *D. discoideum* is phenotypically complex, with social success depending on the specific social partner, it is ultimately a consequence of a simple developmental “decision”: to produce either stalk or spore cells. Stalk and spore cell differentiation is regulated by the production of—and response to—an array of diffusible stalk-inducing factors (StIFs) [9]–[12]. We therefore reasoned that the regulation of StIF production and/or response could potentially be a major determinant of the variation in patterns of spore:stalk allocation observed in this system [6], and potentially the outcomes of social interactions between genotypes. To address this, we first used a modeling approach to investigate the effect that varying StIF production and response (which together are the StIF phenotype) has on patterns of clonal spore allocation. We then extended this model to examine how this variation in StIF production and response, which produce differences in clonal allocation, influences spore allocation during chimeric development and thus social success. This model is then used to examine whether variation among genotypes in StIF production and/or response could explain the patterns of spore allocation observed in clonal and chimeric fruiting bodies.

**Figure 1:**
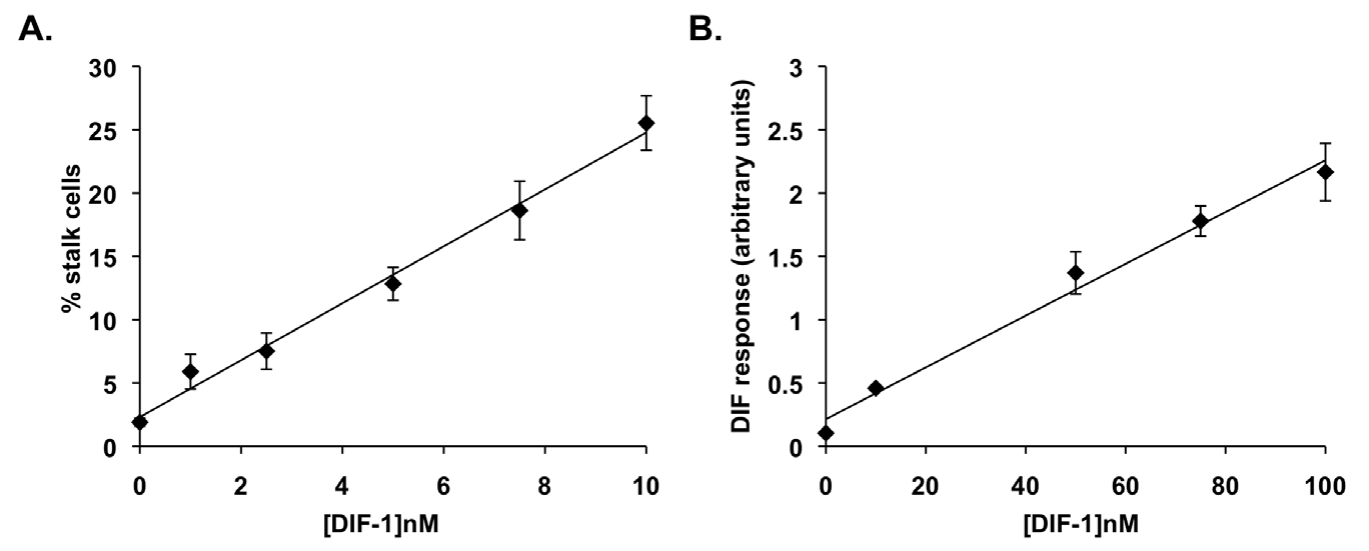
Responses to DIF-1 are linear. (A) Response to the well-characterized StIF, DIF-1, was measured in a monolayer stalk cell induction assay. Cells were plated in a buffered salt solution containing 5 mM cAMP in order to bring cells to competence to respond to DIF-1. After 24 h incubation, cAMP was removed because it is inhibitory to stalk cell differentiation. DIF-1 was then added at varying concentrations for a further 24 h. Stalk cells were counted and expressed as a percentage of total cells. Linear regression *R* ^2^ = 0.878, *p*<0.001. (B) Measurement of induction of a representative prestalk marker gene (*ecmAO-lacZ*) in response to the well-characterized StIF, DIF-1, was measured in a monolayer culture. Cells expressing *ecmAO-lacZ* were plated in monolayer in stalk medium containing 5 mM cAMP in order to bring cells to competence to respond. After 24 h incubation, cAMP was removed and replaced with DIF-1 at varying concentrations for a further 24 h. β-galactosidase activity was measured. Linear regression *R* ^2^ = 0.905, *p*<0.001.

The model is based on two features of the biology of DIF-1, which represents the best characterized StIF: (1) all cells in the aggregate experience the same StIF concentration due to a combination of high diffusibility and constant cell movement [13]–[15] and (2) StIF response is linear within the normal physiological range (Figure 1) [10],[11]. This linear response predicts that differences in StIF production and/or response will lead to changes in allocation patterns.

Because fruiting bodies are comprised of only spore and stalk cells, the spore allocation of genotype *i* with genotype *j* (*a_ij_*) when clonal (*i*  =  *j*) or in chimera (*i* ≠ *j*) is defined simply as the number of cells of genotype *i* that become spores divided by the total number of cells of genotype *i*. Another assumption of the model is that the proportion of spore and stalk cells is governed purely by StIF response (*r*) and production (*s*). Because the response to StIFs is linear, spore allocation of genotype *i* when clonal (*a_ii_*) can be expressed as:
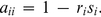
(1)


Note that, because *a_ii_* is a proportion, the values of *s_i_* and *r_i_* are constrained between 0 and 1. Therefore, *s_i_*  = 0 corresponds to no StIF production, whereas *s_i_*  = 1 corresponds to maximum possible StIF production. Likewise, when *r_i_*  = 0 indicates that a genotype has no sensitivity to StIFs, while *r_i_*  = 1 indicates complete sensitivity. It is also important to note that the production parameter (*s_i_*) can also be interpreted as a “potency” parameter, in that it reflects the ability of a signal to induce a developmental change. This potency could, therefore, be due to the amount of signal or the relative ability of that signal to induce differentiation. For simplicity, we call this “production” since there is no evidence that individuals differ in the quality of the StIF signal produced, but we emphasize that this parameter encompasses general signal strength.

**Figure 2:**
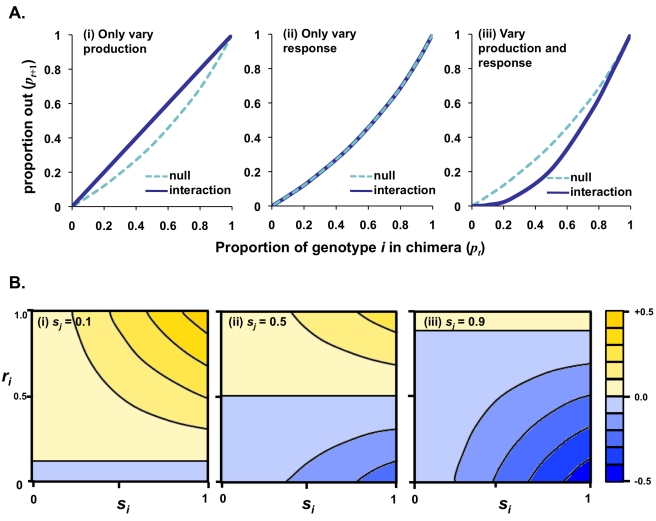
Modeling the effects of varying StIF production and response on behavior. (A) Comparisons of model based chimeric behavior to that predicted from clonal allocation. Each panel shows the frequency of genotype *i* in the chimeric mixture (*p_t_*) with genotype *j* against the frequency of genotype *i* that appears in the sporehead (*p_t_*_+1_) (Equation 3). In each case an example is shown where genotypes *i* and *j* differ in their clonal allocation due to a difference in StIF phenotype (see Equation 1). The “null” line is derived from clonal allocation and assumes no interaction in chimera (Equation 7). The “interaction” line is the outcome predicted by the signal and response model (Equation 11). (i) When genotypes differ in StIF production, but not response, the model predicts facultative shifts in allocation in chimera, but these always result in equal representation in the sporehead (interaction line). This is because each genotype is exposed to the same levels of StIF due to a blending of the extracellular signaling environments and therefore show identical responses. Such behavior has not been observed between natural isolates [1]. (ii) When genotypes differ in StIF response, but not production, there is no change in their spore allocation in chimera (interaction line). This is because the chimeric signaling environment is the same as the clonal one, and so the two show the same response in chimera as they do clonally. (iii) When signal production and responses are both different, genotypes can exhibit differences in both clonal allocation and show shifts in allocation when in chimera similar to the wild isolates. In this example, the differences are such that genotype *i* has lower fitness than expected (interaction line). For example, if a genotype with low production and high response is mixed with a high producer and low responder, then in chimera the low producer experiences higher levels of signal. As a result, it responds to this higher signal level by producing more stalk than predicted by its clonal allocation behavior. (B) Contour “heat” maps showing the range of changes in allocation of genotype *i* when in chimera with *j* (*d_ij_*; Equation 4, see also Equations 1 to 3 and [1]). The heat map represents the change in spore allocation in chimera for the full range of values for response (*r_i_*) to, and production (*s_i_*) of, StIFs for genotype *i* when their chimeric partner, genotype *j*, is a low (i; *s_j_*  = 0.1), medium (ii; *s_j_*  = 0.5), and high (iii; *s_j_*  = 0.9) producer. Self-promotion occurs when the spore allocation increases in chimera, i.e. *d_ij_* is positive (yellow shades), and coercion occurs when spore allocation decreases, i.e. *d_ij_* is negative (blue shades). Note that the StIF response of genotype *j* does not affect the response of its partner (because these are allocation values, not fitness). For all cases, the proportion of genotype *i* (*p_i_*) is 0.5. See Materials and Methods for a full description of the model.

The model also predicts that the spore allocation of *i* when in chimera with *j* will be dependent upon the response and production of *i*, as well as the production of StIFs by *j*. Spore allocation will therefore also depend on the relative proportion of each genotype in the chimera:
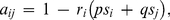
(2)where *p* and *q* are the proportions of *i* and *j*, respectively. This means that there will only be a facultative change in spore allocation in chimera when *s_i_* ≠ *s_j_* (because *r_i_* does not depend on the chimeric partner). To explore this idea, we first derived an expression for the proportion of genotype *i* in the sporehead (*p_t_*_+1_) in terms of StIF response and production:
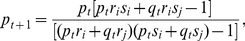
(3)where *p_t_* and *q_t_* are the proportion of genotype *i* and *j* before development (for full development of the model, see Materials and Methods). Equation 3 predicts the representation in the sporehead if the mechanism of interaction is based on StIF phenotypes (“interaction line”) (Figure 2A). This can be compared to the behavior that would be expected from the null hypothesis that there is no interaction and proportions are determined simply by clonal allocation (“null line”) (Figure 2A). Importantly, the model predicts that to generate the patterns of behavior observed in natural isolates [1], both genotypes must vary in StIF response *and* production (Figure 2A).

To extend this idea further we explored the range of facultative behaviors that can be generated by the model. As facultative change (*d_ij_*) is most simply defined as the difference between chimeric and clonal allocation (*a_ij_* − *a_ii_*), it can be expressed in terms of StIF production and response (Equations 1 and 2):
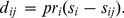
(4)


Therefore, facultative shifts in allocation in chimera compared to clonal are expected to depend upon (a) a genotype's own response to StIF, (b) the difference between a genotype's StIF production and that of its chimeric partner, and (c) the frequency of the two genotypes in the chimera. Using this, we found that the model is sufficient to generate a wide range of facultative behaviors from self-promotion to coercion (Figure 2B).

### A Novel Genetic Selection for Loser Mutants

**Figure 3:**
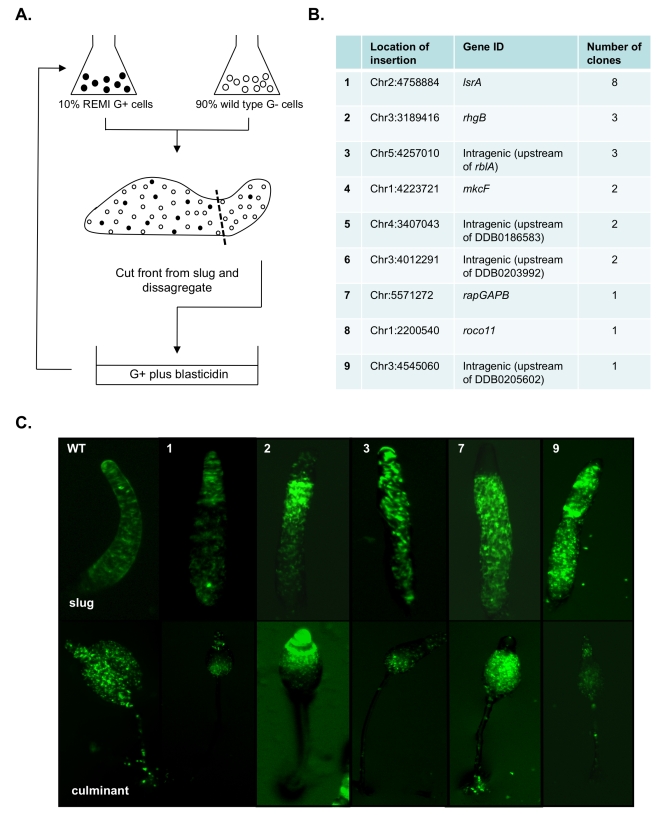
The selection strategy to enrich for “loser” mutants. (A) A pool of ∼1,000 blasticidin resistant mutants was generated by insertional mutagenesis and grown under conditions that bias cells towards the spore cell fate (glucose (G+)). Mutant cells grown under biased conditions (G+) were mixed with an excess of wild type cells. Mixtures were developed to the slug stage and the anterior prestalk region harvested into medium containing blasticidin to kill off wild type cells. This selection strategy was then repeated. (B) Summary of mutants isolated in the screen. We identified the insertion sites from 23 randomly chosen clones from the loser selection. We found that eight of these were insertions within the *lsrA* gene. Other insertional mutants were isolated at lower frequencies. Each mutant was labeled with GFP and their sorting behavior in chimera with wild type was observed. (C) Images of GFP-labeled mutants that exhibit impaired sorting behavior in chimera with wild type. 10% GFP-labeled mutant cells were mixed with 90% unlabelled wild type cells and developed. Their localization in chimera was observed at the slug and culminant stages. A variety of sorting behavior was observed.

**Figure 4:**
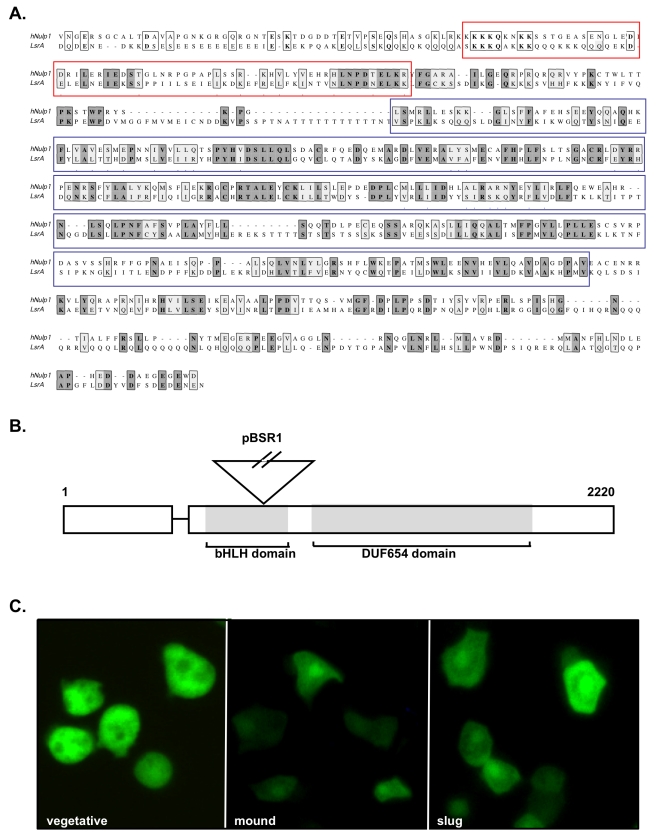
LsrA encodes a putative transcriptional regulator related to human Nulp1. (A) Alignment of human Nulp1 with LsrA. This family is characterized by a conserved DUF654 domain of unknown function (boxed in blue) and the N-terminus shows weak homology to the bHLH DNA-binding and protein-protein interaction domain (boxed in red). Identical residues are highlighted in dark grey and conserved residues in light grey. (B) Structure of the *lsrA* gene to illustrate the position of the insertion cassette. Numbers indicate base pairs. (C) Developmental regulation of LsrA subcellular localization. A LsrA-GFP fusion protein was expressed under the control of a constitutive promoter. LsrA-GFP is evenly distributed throughout vegetative cells but is enriched in the nucleus and at the cell periphery at multicellular stages of development (mound and slug). The LsrA-GFP fusion construct was able to rescue defects associated with the *lsrA* ^−^ mutant (unpublished data).

**Figure 5:**
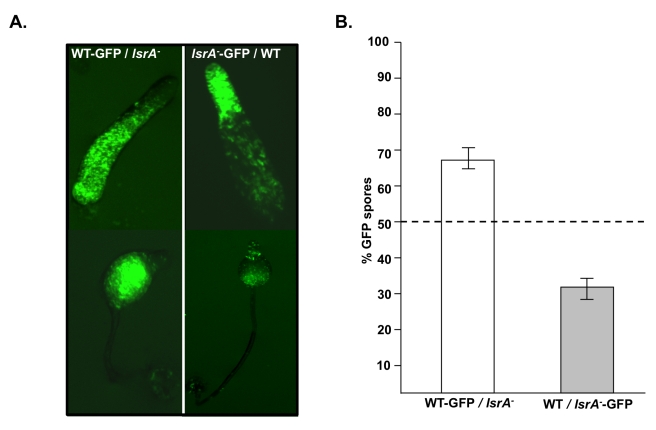
*lsrA* ^−^ behaves as a loser. (A) Localization of different genotypes in chimeric slugs and fruiting bodies. GFP expressing wild type cells are enriched in the prespore and spore regions when mixed with unlabelled *lsrA* ^−^ cells. In contrast, GFP expressing *lsrA* ^−^ cells are enriched in the anterior prestalk region of slugs and prestalk-derived upper and lower cup of fruiting bodies when mixed with unlabelled wild type cells. Homotypic mixes showed an even distribution. (B) Quantification of the contribution of each genotype when labeled to the spore population in chimeric development. Equal proportions of wild type and mutant cells were mixed and developed and spores harvested. GFP-expressing wild type cells are overrepresented in the spore population during chimeric development (1-sample *t* test, *t*_4_ = 7.868, *p* = 0.001) and GFP-expressing *lsrA* ^−^ cells are underrepresented (1-sample *t* test, *t*_3_ = 79.212, *p*<0.001). Dotted line shows proportions in homotypic mixes.

The model predicts that apparently complex “facultative” changes in behavior across interactions can be achieved through changes in developmental signaling in the absence of a recognition mechanism. To test this idea, we firstly devised a novel genetic selection experiment to identify single gene mutations that exhibit altered social behavior wherein they lose in competition (loser mutants). Mutants were enriched that preferentially form prestalk cells at the slug stage of development when mixed with wild type cells (Figure 3A). After six rounds of selection, mutants with disruption of the *lsrA* gene were by far the most strongly overrepresented and therefore chosen for further study (Figure 3B and 3C). The *lsrA* gene is predicted to encode a member of the bHLH family of transcription factors and becomes strongly enriched in the nucleus in developing cells, consistent with a role in the regulation of developmental gene expression (Figure 4). Clonal growth and developmental timing of the *lsrA* ^−^ mutant is identical to wild type (Figure S1). However, as expected, *lsrA* ^−^ mutant cells are over-represented in the prestalk population when developed in chimera with wild type cells (Figure 5A and Figures S2 and S3). Importantly, quantification reveals that mutant cells are, as expected, under-represented in the spore population of chimeric fruiting bodies (Figure 5B).

### Mutation of the *lsrA* Gene Results in Clonal and Chimeric Changes in Behavior

**Figure 6:**
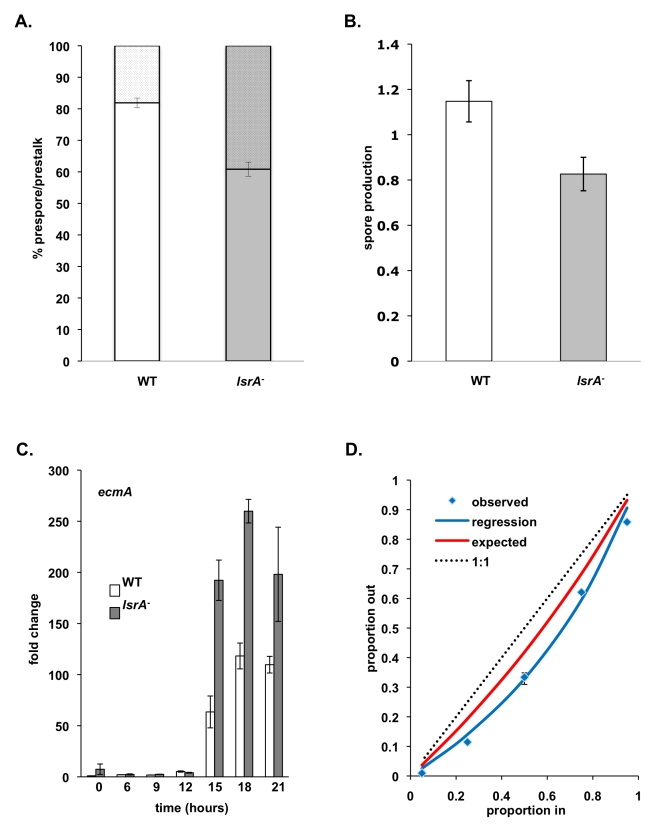
*lsrA* ^−^ exhibits differences in both clonal and chimeric spore allocation. (A) Spore:stalk ratios. Cells from dissociated slugs were stained with a prespore cell–specific antibody and the percentage of stained cells measured. The spore:stalk ratio of wild type is 80:20±1.5, whereas the spore:stalk ratio of *lsrA* ^−^ is 60:40±2.3 (*t* test, *t*_16_ = 22.714, *p*<0.001). (B) Total spore production (measured as the relative output number of spores compared to the input number of amoebae) after fruiting body formation in *lsrA* ^−^ is reduced compared to wild type cells during clonal development (*t* test, *t*_22_ = 9.682, *p*<0.001). (C) Expression of the prestalk-specific gene, *ecmA*, was measured by quantitative PCR in wild type and *lsrA* ^−^ mutant cells during development. Expression is higher in *lsrA* ^−^ cells compared to wild type. Results are averages and standard deviations of three biological replicates, where each replicate was performed in triplicate. (D) Quantification of the contribution of *lsrA* ^−^ cells to chimeric fruiting bodies when mixed at different input frequencies. Dotted line shows a fair interaction in which both genotypes contribute equal numbers to spores. Red line (calculated using the fixed allocation model [1]) shows contribution of *lsrA* ^−^ cells to spores predicted by fixed allocation. Blue squares show the observed contribution of *lsrA* ^−^ cells to the sporehead, with best fit regression line (blue line, least-squares differences, *F*_1,4_ = 409.8, *p*<0.001), demonstrating a shift in behavior in chimera that deviated from that expected based on clonal allocation.

We next tested whether the *lsrA* ^−^ mutant exhibits a difference in clonal spore allocation compared to wild type and shows a shift in allocation when in chimera [1]. During clonal development, the *lsrA* ^−^ mutant was found to produce fewer prespore cells at the slug stage (Figure 6A) and fewer spores after fruiting body formation (Figure 6B), as well as exhibiting higher levels of prestalk gene expression (Figure 6C), thus demonstrating an altered spore allocation strategy. If differences in spore allocation observed in clones account for the differences in chimeric spore production, clonal spore allocation values should predict the relative fitness of the two genotypes in chimera (Figure 6D; “expected” line). To test this idea, *lsrA* ^−^ mutant cells were mixed with wild type cells at different input frequencies and the relative number of spores of each genotype quantified. Surprisingly, the relative number of *lsrA* ^−^ mutant spores in chimeric fruiting bodies was always lower than that predicted by a fixed strategy alone, demonstrating that mutation of a single gene can lead to shifts in behavior in chimera (Figure 6D; “regression” line).

### Measurements of StIF Production and Response in the lsrA Mutant Predict Clonal and Chimeric Behavior

**Figure 7:**
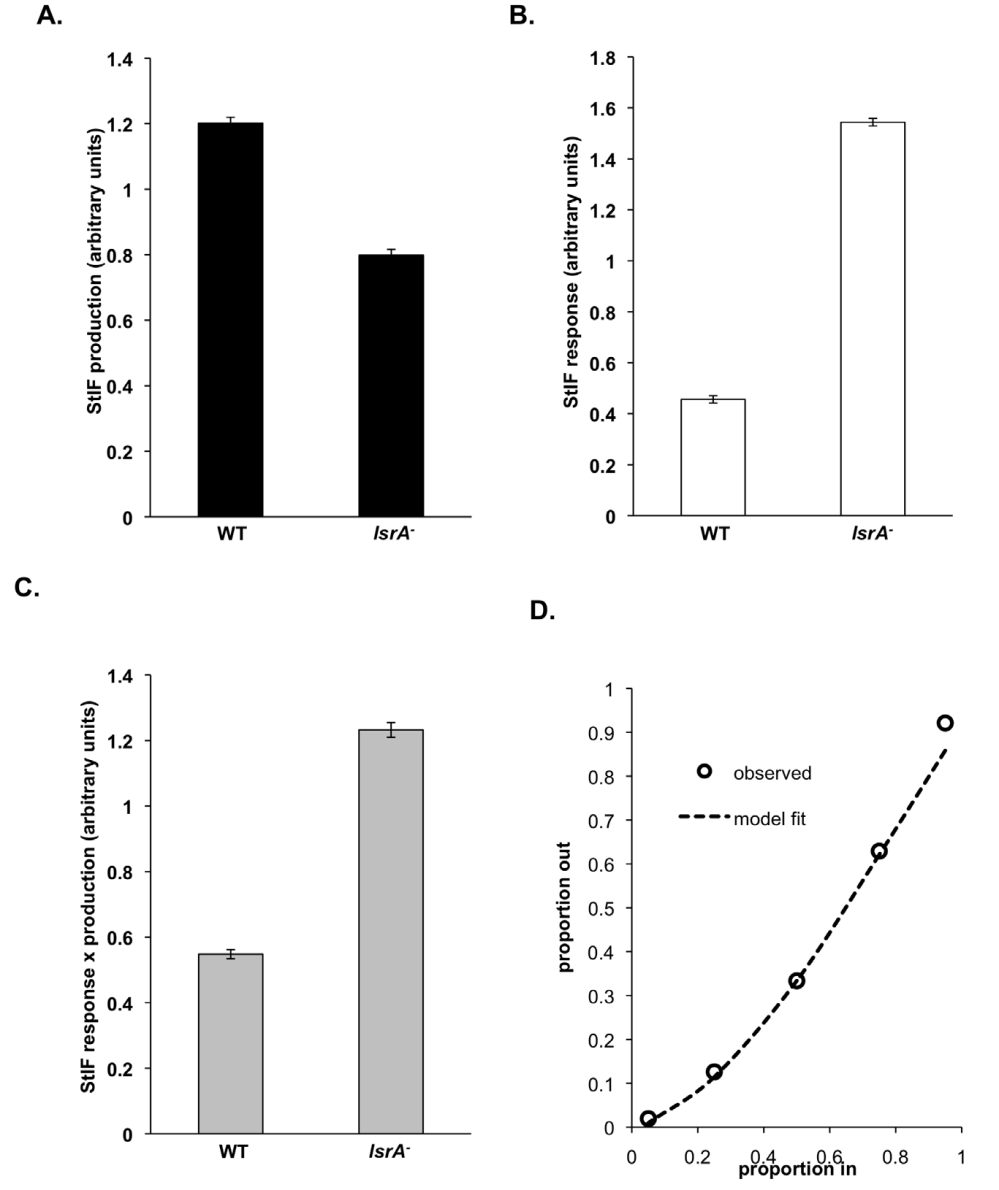
Responses to—and production of—StIF can predict clonal and chimeric allocation of the *lsrA* ^−^ mutant. (A) Induction of *ecmAO*-lacZ by StIFs collected from wild type and *lsrA* ^−^ cells. Cells expressing *ecmAO*-lacZ were developed in monolayer and gene expression induced by StIFs collected from strains as indicated. Induction by *lsrA* ^−^ StIF was 0.67 times less than wild type StIF (*t* test, *t*_14_ = 11.592, *p*<0.001). (B) Induction of *ecmAO*-lacZ in wild type and *lsrA* ^−^ cells by StIF. Cells expressing *ecmAO*-lacZ were developed in monolayer and gene expression induced by StIF. The response of *lsrA* ^−^ cells was 3.04-fold higher compared to wild type cells (*t* test, *t*_14_ =  −50.68, *p*<0.001). (C) Multiplying the response measurement by the production measurement predicts that the clonal stalk allocation of the *lsrA* ^−^ mutant is 2.10 times greater than wild type. (D) The model (Equation 3) can predict the fitness curve of the *lsrA* ^−^ mutant in chimera with wild type (least-squares best-fit; *F*_1,4_ = 346.1, *p* = 0.0003; see Materials and Methods). This shows that the model not only successfully predicts general patterns but can also generate quantitative predictive data with some precision.

If the shifts in clonal and chimeric spore allocation behavior seen in the *lsrA* ^−^ mutant are generated through changes in StIF production and response, as our model predicts, both must differ in the wild type and mutant. To measure StIF production, conditioned medium containing StIFs was isolated from developing wild type or *lsrA* ^−^ mutant cells and tested for its ability to induce the expression of representative prestalk marker genes. *lsrA* ^−^ conditioned medium was a less potent inducer than wild type (Figures 7A and S4). In contrast, when the responsiveness of each strain was compared, the *lsrA* ^−^ mutant was found to be more responsive (Figures 7B and S4). Consequently, as the model predicts, the *lsrA* ^−^ mutant differs from wild type in both StIF production *and* responsiveness.

Most importantly, relative StIF production and response measurements can be used to test whether the model predicts clonal spore allocation and the shift in allocation in chimera. The value of response × production (Equation 1) for the *lsrA* ^−^ mutant is 2.1 times higher than wild type (Figures 7C and S4), suggesting a 2.1-fold difference in prestalk cell number. Consistent with this prediction, measurements of prestalk cell number in dissociated slugs reveal a 2.0-fold difference between wild type (20.0% ±1.5%) and *lsrA* ^−^ mutant (39.7% ±2.3%) (Figure 6A). Secondly, we tested whether the model can predict the shift in spore allocation observed in chimera. Using the response and production measurements, the model accurately predicts the spore:stalk allocations of both strains when chimeras formed from different frequencies (Figure 7D and Materials and Methods).

### StIF Production and Response Can Also Predict Social Interactions Across a Range of Naturally Occurring Wild Isolates

**Figure 8:**
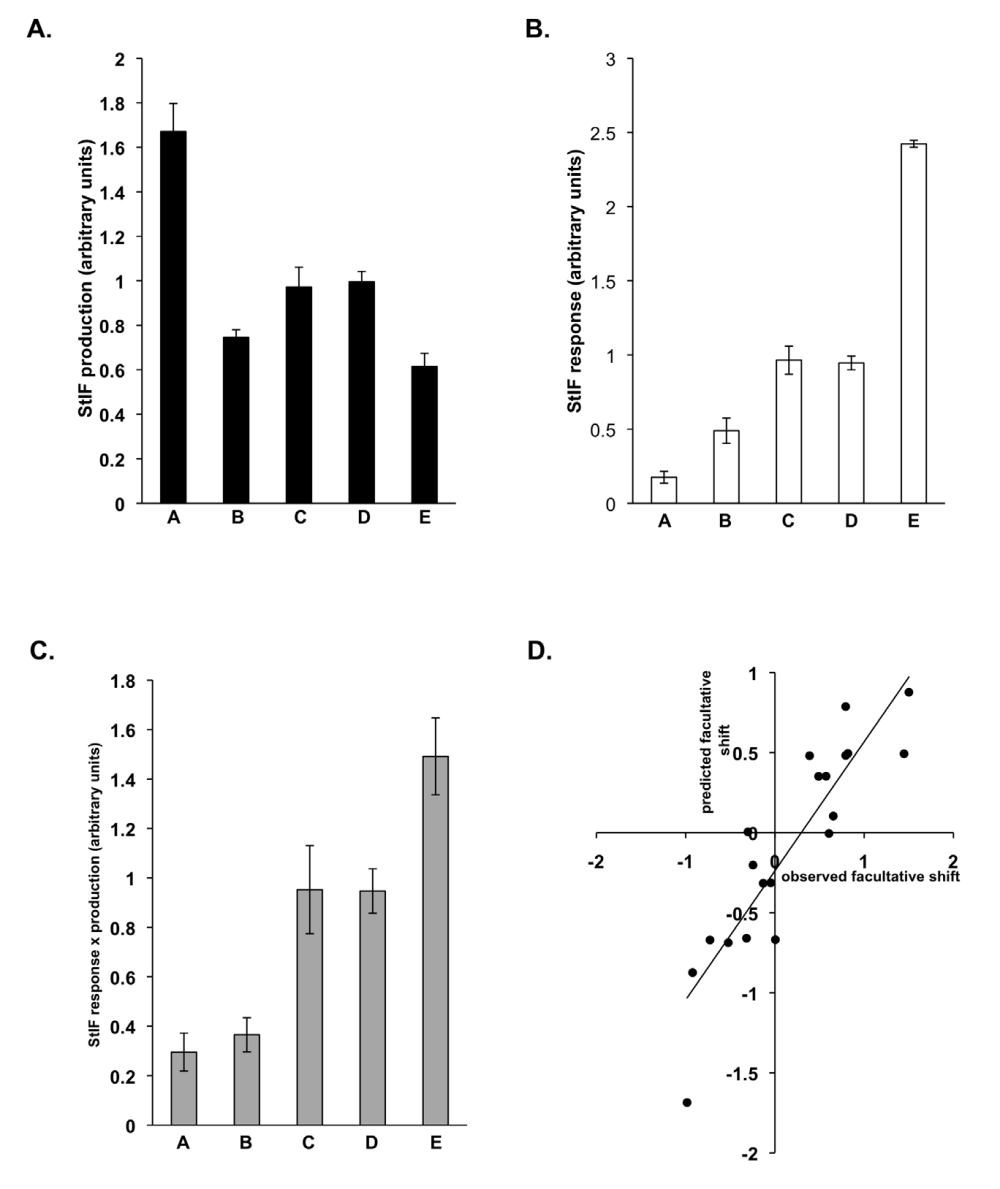
Responses to—and production of—StIF can predict clonal and chimeric allocation patterns of the wild isolates. (A) Induction of *ecmAO*-lacZ by StIFs collected from natural isolates A, B, C, D, and E. Cells expressing *ecmAO*-lacZ were developed in monolayer and gene expression induced by StIFs collected from isolates as indicated. Induction varied dramatically across the five isolates (one-way ANOVA, *F*_4,10_ = 27.026, *p*<0.001). (B) Induction of *ecmAO*-lacZ in natural isolates A, B, C, D, and E by StIF. Cells expressing *ecmAO*-lacZ were developed in monolayer and gene expression induced by StIF. The response varied dramatically across the five isolates (one-way ANOVA, *F*_4,10_ = 4.916, *p* = 0.016). (C) Multiplying the response measurement by the production measurement can predict the hierarchy of stalk allocation for the natural isolates. (D) Correlation of observed facultative shifts in allocation of natural isolates in chimera compared to those predicted by the model (see Equation 12) (Pearson correlation *r*_18_ = 0.8924, *p*<0.001). A positive value indicates an increase in allocation (promotion) and a negative value indicates a decrease in allocation (coercion).

We next tested whether differences in StIF production and responsiveness could also account for variation in the behavior of five genotypes isolated from a natural population, which are known to exhibit different clonal spore allocations and partner-dependent shifts in behavior (chimeric spore allocation) [1]. The five isolates show significant differences in StIF production, with almost a 3-fold difference between the highest and lowest producer (Figures 8A and S5). Furthermore, when the responsiveness of each isolate was measured, significant differences were apparent with almost a 15-fold difference between the highest and lowest responder (Figures 8B and S6). Therefore, naturally occurring *D. discoideum* isolates exhibit, as predicted, widespread natural variation in StIF production and response.

We next tested whether these differences in StIF production and response could account for the differences in clonal spore production (i.e. fixed strategies) that are responsible for the linear social dominance hierarchy seen in these isolates, with isolate *A* producing the least stalk and isolate *E* the most stalk [1]. We find that differences in StIF responsiveness alone are almost sufficient to account for this hierarchy, whereas no correlation is seen between the hierarchy and relative StIF production (Figure 8A and 8B). Most importantly, however, when values of StIF production and response are considered together (as in Equation 1), the hierarchy is faithfully reproduced (Figure 8C). The spore allocations predicted by the model using these measurements closely match the observed values (Pearson correlation; *r_3_* = 0.942, *p* = 0.017) [1]. Finally, we tested whether these values could account for the changes in spore allocation behavior that genotypes exhibit across different chimeric combinations [1], where genotypes show social context-dependent (partner-specific) changes in allocation behavior. We found that these partner-specific responses predicted by the model (using the estimated StIF phenotype of each genotype) accurately predict (Pearson correlation; *r_18_* = 0.8924, *p*<0.001) the observed spore allocation behavior in chimeras previously described (see Materials and Methods) (Figure 8D) [1], demonstrating that the StIF signaling system appears to account for the complex social context-dependent shifts in social behavior that have been reported for *D. discoideum.*

## Discussion

Our findings suggest that seemingly complex social behavior can have a relatively simple underlying developmental mechanism, in this case the regulation of signal production and response. As a result, social behavior can be accurately predicted from measurements of the signal production and response phenotype using a simple linear model. This is at odds with the notion that partner-specific responses would require some partner recognition system for genotypes to invoke a partner-specific strategy [1],[7],[8],[16]. Indeed, we find that apparent partner-specific responses occur because the signaling system is “interactive” or epistatic, where the response of a genotype in a social interaction depends on both its own signal sensitivity and the signal production of the social partner relative to its own production (Equation 4). As a result, genotypes respond differentially to the same social partner because they differ in either their sensitivity to StIFs or their own StIF production (or both).

Our results have implications for the definition of what has been described as fixed and facultative behavior in this system (and more generally). Specifically, we demonstrate that apparently facultative outcomes of interactions do not necessarily imply facultative changes when viewed at a mechanistic level. In this case, fixed clonal differences in social signals result in seemingly unpredictable facultative outcomes. Therefore, “social strategies” may be manifested largely as a set of knowable parameters related to StIF production and response, making patterns of behavior predictable in this system. Previous work has characterized patterns of genetic variation in this system in terms of the genetic control of the outcome of interactions by partitioning variation in allocation patterns into direct genetic effects, attributable to the genotype of the focal genotype; indirect genetic effects, attributable to the genotype of the social partner genotype; and genotype-by-genotype (G×G) epistasis, attributable to the specific combination of genotypes in an interaction [6]. Our model is consistent with these ideas and would suggest that direct genetic effects are largely determined by signal sensitivity but also partly by signal production (since individuals always determine part of the signaling environment they experience), while indirect genetic effects would be determined entirely by signal production, where genotypes influence each other as a function of the amount of StIFs that they produce. The G×G epistasis would, therefore, be a consequence of the interactive nature of the system, where the indirect genetic effect depends on the sensitivity of the focal genotype and on the difference in signal production of the interacting genotypes (cf. Equation 4).

We have found that disruption of a single gene, *lsrA* ^−^, is sufficient to generate changes in both clonal and chimeric behavior. This is because the *lsrA* gene exerts pleiotropic effects on both signal production and response. One explanation for these wide-ranging effects may come from the finding that *lsrA* encodes a protein with homology to bHLH family transcription factors, which could potentially regulate the expression of genes required for both normal signal production and response. Indeed, it has previously been demonstrated that production and response of DIF-1, a well-characterized example of a StIF, are indeed coupled, with increased DIF-1 response resulting in decreased DIF-1 biosynthesis and increased DIF-1 breakdown [17],[18]. One consequence of this idea, however, is that it would be expected to lead to runaway social evolution, where there is constant selection for increased signal production and reduced response, whereby genotypes coerce others to produce stalks, while simultaneously decreasing sensitivity, thereby decreasing the ability of individuals to be exploited by the social signal. Such a directional runaway process predicts the system would either be devoid of standing genetic variation in signal production and response because variation would be rapidly depleted by strong social selection or would only contain variation that shows antagonistic pleiotropy (which, in this case, would be associated with a positive correlation in pleiotropic effects where those that are high producers are high responders and vice versa).

Despite this expectation, however, we find that natural isolates show a wide range of signal productions and sensitivities, with an overall negative correlation between signal production and response (i.e. those that produce more signals are less sensitive to it) among natural isolates. These isolates therefore follow the same basic pattern seen for the single *lsrA* gene mutation. This observation suggests that pleiotropic effects of mutations may generally be negative due to some feature of the biology of the system. However, it is also possible that much of the variation in the StIF system is not an outcome of selection but, rather, is largely an outcome of the random processes of mutation and drift. The influence of social selection in determining patterns of variation could be restricted due to the fact that chimerism is limited [19] and the social phase only occurs rarely compared to the intervening free-living generations, both of which reduce the effectiveness of selection for success in chimera (leading to the presence of more variation simply because of weak social selection) [20]. The latter of these will also reduce the impact of natural selection (i.e. “non-social” selection occurring among clones) on patterns of variation for clonal development. Because natural selection must favor the successful production of a stalk that holds aloft a sporehead, there is a potential trade-off between dispersal, favoring a larger stalk, and fecundity, favoring a larger sporehead. Therefore, it is possible that the negative correlation between StIF production and response observed is determined by such a natural selection trade-off. In this scenario, variation occurs because the fecundity-dispersal trade-off leads to similar fitness for a range of different spore allocation values, producing weak selection on specific allocation values but selection for the coordination of signal production and response through negative pleiotropy.

Importantly, although our studies reveal that complex behavior can be generated by a simple system output, it seems likely that the underlying pathways regulating signal production and response may be more complex [21]. For example, many genes can potentially modulate StIF production (e.g. biosynthesis, breakdown) and response (receptor, signal transduction, transcriptional output). *lsrA* is likely to be just one of many such genes inputting into pathways and networks that ultimately determine the “summary statistics” of signal production and sensitivity. Evolution of social strategies, therefore, would operate through these potentially diverse underlying pathways while manifesting themselves at the level of the simple interaction of the StIF system. But the fact that interactions may be largely governed by the interface of StIFs suggests that there is a constraint on the patterns of social behavior we expect to observe. The simple linear model of the StIF system is expected to result in a linear (transitive) social dominance hierarchy. Such linearity has been observed in this system [1],[22] and, therefore, may reflect a developmental constraint on the evolution of the dominance hierarchy structure imposed by the linearity of the StIF system itself.

Taken together, our studies suggest that even though complex and seemingly unpredictable outcomes can result from social interactions, they can be governed by a set of simple rules. Therefore, our studies provide a novel solution to the generation of complex (apparently unpredictable) social behavior, in this case based on the production and response to social signals. This result is not, however, at odds with the occurrence of biological complexity in this system but, rather, implies that the underlying complexity of gene networks is ultimately played out in the social arena through a simplified interface that dictates the result of social encounters. We therefore suggest that our understanding of the evolution and maintenance of social behavior will be greatly aided by defining basic rules governing interactions, as much as identifying the genes and pathways underlying social behavior.

## Materials and Methods

### Cell Growth and Maintenance

Lab strains (*AX4*) and North Carolina wild isolates [1] were maintained in liquid culture in HL5 medium or in association with *Klebsiella aerogenes* bacteria. Reporter gene plasmids were transformed by electroporation [23].

### REMI Mutagenesis and Mutant Isolation

For REMI mutagenesis [24], *AX4* cells were grown to 2×10^6^ cells/ml in liquid HL5 medium. Cells were resuspended at 1×10^7^ cells/ml in electroporation buffer (10 mM Na_2_HPO_4_, 50 mM sucrose, pH 6.1) and mixed with 10 µg of *BamHI* linearized pBSR1 and 10 units of *DpnII* restriction enzyme. Cells were electroporated at 1.0 kV and 3 µF before plating. Cells were selected in 10 µg/ml blasticidin.

For prestalk sorting mutant selection, a pool of 1,000 insertional mutants was grown in shaken culture at 22°C in HL5 medium in the presence of glucose before developing in chimera at a 10:90 ratio with wild type *AX4* cells grown in the absence of glucose. Cells were developed on sterile KK2 plates containing 1.5% L28 agar (Oxoid) until the slug stage (14–16 h), at which point the anterior 25% of the slug was cut off using a sterile sharpened insect pin. Cells were disaggregated in disaggregation buffer (20 mM EDTA in KK2) and grown in filter sterilized HL5 medium containing 86 mM glucose and 10 µg/ml blasticidin in order to kill off wild type *AX4* cells. The surviving blasticidin resistant cells were then transferred to shaken culture in HL5 medium containing 86 mM glucose and subjected to six rounds of selection. Plasmid insertion sites were identified by inverse PCR [25]. 10 µg genomic DNA was digested with *RsaI* and purified. For the ligation, 5 µg of the digested DNA was added to 40 µl of 10× T4 DNA ligase buffer and 2 µl of T4 DNA ligase in a total reaction volume of 400 µl. The ligated DNA was precipitated and subjected to inverse PCR using primers specific to a region on the actin 15 promoter of the insertion vector. The products of the PCR reaction were purified and sequenced.

For the disruption of the *lsrA* gene, a 7 kb genomic fragment including insertion cassette was amplified by PCR from the *lsrA* locus in the *lsrA* REMI mutant isolated from the screen. The linearized construct was transformed into *AX4* cells by electroporation followed by blasticidin selection and confirmation of gene disruption by PCR.

### Transformation of Wild Isolates with lacZ Reporter Genes

Wild clones were grown in association with *Klebsiella aerogenes* and co-transformed with actin15-RFP and *lacZ* reporter plasmids by electroporation [23]. Clones were selected in HL5 medium containing 20 µg/ml G418 for 1 wk before plating out clonally in association with bacteria. Fluorescent clones were picked and tested for *lacZ* expression.

### Quantification of Fixed and Facultative Strategies

Total spore production and relative number of GFP labeled spores was measured in strains developed clonally or in chimera [1]. To detect changes in sorting behavior, GFP labeled strains were mixed with unlabeled cells and examined. For measurement of prespore:prestalk ratio, dissociated slug stage cells were fixed and stained with prespore-specific anti-psv antibody [26].

### Characterization of Marker Gene Expression During *lsrA* ^−^ Mutant Development

Cell type–specific marker transformants were selected in 20 µg/ml G418. For development, cells in exponential growth phase were harvested and washed before plating at a density of 6.4×10^6^ cells/cm^2^ on KK2 (16.1 mM KH_2_PO_4_, 3.7 mM K_2_HPO_4_) plates in 1.5% purified agar. For quantification of *lacZ* expression, 1×10^7^ cells from slugs and culminants were lysed in 100 µl lysis buffer (100 mM HEPES, 1 mM MgSO_4_, 2% Triton X-100, 5 mM DTT, pH 8.0) and the protein concentration measured against a BSA standard curve. The amount of β-galactosidase enzyme activity per µg of protein was measured by adding a known amount of protein to 100 µl lysis buffer containing 2 mM CPRG (Roche). β-galactosidase enzyme activity was monitored by measuring the color change at 550 nm. For quantification of cell type–specific gene expression, cDNA was obtained from cells throughout development. Gene expression was measured using qPCR [27].

### Measuring StIF Production and Response

For the collection of conditioned medium and induction of *lacZ* reporter genes, cells were grown in the presence of *Klebsiella aeorogenes*. Mid-log phase cells were harvested, washed, and resuspended at 1×10^5^ cells/ml in stalk medium (10 mM MES (pH 6.2), 1 mM CaCl_2_, 2 mM NaCl, 10 mM KCl, 200 µg/ml streptomycin sulphate) containing 5 mM cAMP. Conditioned medium was collected from plates after 20 h incubation. For the induction of *lacZ* [28], cells were incubated for a further 4–6 h with or without StIF or DIF-1. Cells were then lysed in 100 µl lysis buffer (100 mM HEPES, 1 mM MgSO_4_, 2% Triton X-100, 5 mM DTT, pH 8.0) containing 2 mM CPRG. β-galactosidase enzyme activity was monitored by measuring the color change at 550 nm. To obtain overall production values, response data from all genotypes were pooled and scaled by the average in order to remove differences in responsiveness. To obtain overall response values, production data from all genotypes were pooled and scaled by the average in order to remove differences in production. Experiments were performed three times.

### Modeling of StIF Production and Response

Because fruiting bodies are comprised of spores and stalk cells only, the spore allocation of genotype *i* (*a_ij_*) when clonal (*i*  =  *j*) or in chimera (*i* ≠ *j*) is defined simply as the number of cells of genotype *i* that become spores divided by the total number of cells of genotype *i*.

### Clonal Allocation and Null Expectations

The behavior of a genotype when clonal can be considered the “fixed” component of its social strategy. As such, it can also be used to determine the “null” behavior of genotypes in chimera under the assumption that there is no facultative change in allocation when in chimera (i.e. that clonal behavior predicts behavior in chimera).

We assume that the proportion of cells of genotype *i* that become spore or stalk is determined by the level of StIF present and genotype *i*'s response to that signal (*r_i_*). When clonal, the StIF level is determined solely by the signal production of the genotype itself (*s_i_*), and therefore, clonal allocation of cells to spore is defined as:
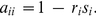
(5)


Note that, because *a_ii_* is a proportion, the values of *s_i_* and *r_i_* are constrained between 0 and 1. Therefore, *s_i_*  = 0 corresponds to no StIF production, whereas *s_i_*  = 1 corresponds to maximum possible StIF production. Likewise, when *r_i_*  = 0 indicates that a genotype has no sensitivity to StIFs, while *r_i_*  = 1 indicates complete sensitivity.

Clonal spore allocation can be used to calculate the expected null fitness (*w_ij_*_(*e*)_) or “social success” of genotype *i* in competition with *j*:
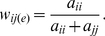
(6)


These fitness values are relative such that the higher spore allocator would have the higher fitness in a chimera and *w_ij_*_(*e*)_ + *w_ji_*_(*e*)_  = 1. Therefore, *w_ij(e)_* is a “coefficient of social success” because it is a constant that determines the proportion of genotype *i* after development (*p_t_*_+1(*e*)_) with *j* from any initial frequency (*p_t_*):
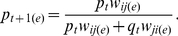
(7)


Because Equation 7 gives the proportion of genotype *i* present in the sporehead of a chimeric mixture in the absence of facultative social behavior by either genotype, it therefore represents the null (non-facultative) “expected” lines in Figures 2E and 3A.

### Chimeric Allocation and Expected Interactions

When in chimera, the StIF level is determined by the proportional representation of the two genotypes in the chimera and their individual levels of StIF production. Therefore following Equation 5, the spore allocation of genotype *i* in chimera with genotype *j* is:
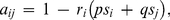
(8)where *p* and *q* are the proportions of *i* and *j*, respectively. This means that when *s_i_* ≠ *s_j_* there will be a facultative change in spore allocation chimera. When the behavior of genotypes is different to that expected under the null model, the behaviors are referred to as “interacting” behaviors. Following the conventions of Equation 6, the actualized fitness of *i* with *j* (*w_ij_*) is:
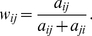
(9)


Substituting Equation 8 into 9 gives an expression for *w_ij_* in terms of StIF response and production:
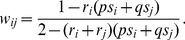
(10)


This means that the model predicts that the fitness of *i* with *j* will be frequency dependent. Following Equation 7, observed proportion of genotype *i* (*p*_(*t*+1)_) within the sporehead after development with genotype *j* is given by:
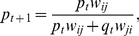
(11.1)which, in terms of StiF response and production, is:
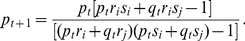
(11.2)


Equation 11.2 is therefore the equation for the “interacting” lines in Figures 2E and 3A.

The model also predicts changes in behavior in chimera (*d_ij_*), defined simply as *a_ij_* − *a_ii_* (i.e. deviation in allocation when in chimera compared to that seen clonally), as a function of StIF response and production:
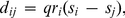
(12)which demonstrates that shifts in allocation in chimera are expected to depend upon (a) a genotype's own response to StIF, (b) the difference between a genotype's StIF production and that of its chimeric partner, and (c) the frequency of the two genotypes in the chimera. See Figure 3B for the expected range of facultative behaviors.

### Estimating the Stalk Allocation of *lsrA* ^−^

If the estimate of spore allocation of the wild type is 80% and the mutant makes 0.72× the spores as wild type (Figure 2C), then the spore allocation of wild type can be estimated to be 0.8×0.72 = 0.576. This converts to a stalk allocation for wild type and mutant of 0.2 and 0.424, respectively, i.e. the stalk allocation of *lsrA* ^−^ should be 2.12× that of wild type.

### Generating a Fitness Curve From Response and Production Estimates

To generate the fitness curves in Figure 4D, the proportion of *lsrA* ^−^ spores within the sporehead after development with wild type (*p_t_*_+*1*_), i.e. the “model fit” line, was calculated using Equation 11.2. The fitness of the mutant (*w_lsr.wt_*) was frequency dependent as predicted in Equation 10 and declined with increasing frequency. Strikingly, the model presented here fit the observed data very well (least-squares best-fit; *F*_1,4_ = 346.1, *p* = 0.0003) and shows that the model not only successfully predicts general patterns but can also generate quantitative predictive data with some precision. Although fitness was frequency dependent, the model best fit and the fixed fitness model were statistically indistinguishable (least-squares best-fit; *F*_1,4_ = 409.8, *p* = 0.0003).

### Predicting the Chimeric “Facultative” Response of Natural Isolates

The spore allocation of each genotype (*a_ij_*) in every pair was calculated in the same way as described above with the mutant and wild type (Equation 11.2), using the estimates for *r_i_* and *s_i_* for the natural isolates (Figure 4E and 4F). So that the expected chimeric behavior generated from the model could be directly compared to the observed behavior [1], *a_ij_* was calculated when genotypes were in equal proportions only. Facultative change was calculated using Equation 12, where a value greater than zero means that a genotype increased its spore allocation in chimera (i.e. it self-promoted) and a value less than zero means that the genotype's spore allocation decreased in chimera (i.e. it was coerced). We found the model's predicted social behavior to be highly correlated with observed data (Figure 4H; Pearson correlation: *r_18_* = 0.8924, *p*<0.001) [1].

## Supporting Information

Figure S1 *lsrA* ^−^ does not exhibit obvious defects in developmental morphology or timing. *lsrA* ^−^ mutant and wild type cells were developed on non-nutrient agar for the times indicated. Both strains had reached equivalent stages at each time point.
(TIF)

Figure S2 *lsrA* ^−^ exhibits general defects in prestalk cell differentiation when developed in chimera at slug stage. To test which prestalk cell types were affected in the *lsrA* ^−^ mutant, wild type and *lsrA* ^−^ mutant cells were transformed with lacZ markers that drive expression in each of the major prestalk (*ecmA*, *ecmO*, *ecmAO*, and *ecmB*) and prespore (*psA*) cell types. Strains expressing cell type–specific markers were mixed in chimera in a 10:90 ratio with unlabelled cells and relative expression assessed qualitatively and quantitatively at the slug stage. Wild type or *lsrA* ^−^ cells were transformed with cell-specific reporter genes. Clear differences were found in the expression of all prestalk cell-specific markers (*ecmA*, *ecmO*, *ecmAO*, and *ecmB*), although prespore cell-specific markers (*psA*) appear to be less affected. The expression of wild type prestalk markers was lower when mixed with a majority of mutant cells compared to when mixed with a majority of wild type cells. In contrast, the expression of mutant prestalk markers was higher when mixed with a majority of wild type cells compared to when mixed with a majority of mutant cells. To quantify this observation, the level of *lacZ* expression in heterotypic slugs was normalized to *lacZ* expression during homotypic development. The expression of wild type prestalk cell markers decreased when in chimera with mutant cells, whereas the expression of mutant prestalk cell markers increased when in chimera with wild type cells. The expression of the prespore marker showed the opposite pattern. The expression of wild type prespore marker increased when in chimera with mutant cells, whereas the expression of mutant prespore marker decreased when in chimera with wild type cells. Results are averages and standard deviations of three biological replicates, where each replicate was performed in triplicate.
(TIF)

Figure S3 *lsrA* ^−^ exhibits general defects in prestalk cell differentiation when developed in chimera at culminant stage. To test which prestalk cell types were affected in the *lsrA* ^−^ mutant, wild type and *lsrA* ^−^ mutant cells were transformed with lacZ markers that drive expression in each of the major prestalk (*ecmA*, *ecmO*, *ecmAO*, and *ecmB*) and prespore (*psA*) cell types. Strains expressing cell type–specific markers were mixed in chimera in a 10:90 ratio with unlabelled cells and relative expression assessed qualitatively and quantitatively at the culminant stage. Wild type or *lsrA* ^−^ cells were transformed with cell-specific reporter genes. Clear differences were found in the expression of all prestalk cell-specific markers (*ecmA*, *ecmO*, *ecmAO*, and *ecmB*), although prespore cell-specific markers (*psA*) appear to be less affected. The expression of wild type prestalk markers was lower when mixed with a majority of mutant cells compared to when mixed with a majority of wild type cells. In contrast, the expression of mutant prestalk markers was higher when mixed with a majority of wild type cells compared to when mixed with a majority of mutant cells. To quantify this observation, the level of *lacZ* expression in heterotypic slugs was normalized to *lacZ* expression during homotypic development. The expression of wild type prestalk cell markers decreased when in chimera with mutant cells, whereas the expression of mutant prestalk cell markers increased when in chimera with wild type cells. The expression of the prespore marker showed the opposite pattern. The expression of wild type prespore marker increased when in chimera with mutant cells, whereas the expression of mutant prespore marker decreased when in chimera with wild type cells. Results are averages and standard deviations of three biological replicates, where each replicate was performed in triplicate.
(TIF)

Figure S4 *lsrA* ^−^ cells exhibit differences in the responses to—and production of—StIFs. (A) Induction of *ecmB*-lacZ in wild type and *lsrA* ^−^ cells by StIF. Cells expressing *ecmB*-lacZ were developed in monolayer and gene expression induced by StIF. The response of *lsrA* ^−^ cells was 5.5-fold higher compared to wild type cells (*t* test, *t*_4_  =  14.625, *p*<0.001). (B) Induction of *ecmB*-lacZ by StIFs collected from wild type and *lsrA* ^−^ cells. Cells expressing *ecmB*-lacZ were developed in monolayer and gene expression induced by StIFs collected from strains as indicated. Induction by *lsrA* ^−^ StIF was 0.38 times less compared to wild type StIF (*t* test, *t*_4_  =  20.372, *p*<0.001). (C) Multiplying the response measurement by the production measurement can predict that the clonal stalk allocation of the *lsrA* ^−^ mutant is 2.12 times greater than wild type.
(TIF)

Figure S5 Natural isolates exhibit differences in the production of StIF. Induction of *ecmAO*-lacZ in natural isolates by StIF collected from each natural isolate. Cells of one isolate (indicated in the upper left-hand corner of each graph) expressing *ecmAO*-lacZ were developed in monolayer and gene expression measured in response to StIF collected from each isolate. Natural isolates vary dramatically in their production. Data are expressed as fold change in expression compared to no StIF control and are the average of three biological replicates. Significant differences in induction between strains were tested for using one-way ANOVAs.
(TIF)

Figure S6 Natural isolates exhibit differences in the responses to StIFs. Induction of *ecmAO*-lacZ in natural isolates by StIF collected from each natural isolate (indicated in the upper left-hand corner of each graph). Different isolates expressing *ecmAO*-lacZ were developed in monolayer and gene expression measured in response to StIF from a single isolate. Natural isolates vary dramatically in their responsiveness, however the relative responses to each StIF from each isolate are comparable. Data are expressed as fold change in expression compared to no StIF control and are the average of three biological replicates. Significant differences in induction between strains were tested for using one-way ANOVAs.
(TIF)
