# Supplementary figures and images for: A Simple Mechanism for Complex Social Behavior
Source: PLoS Biol. 2011 Mar 29;9(3):e1001039. doi: 10.1371/journal.pbio.1001039 (PMC3066132; doi:10.1371/journal.pbio.1001039)

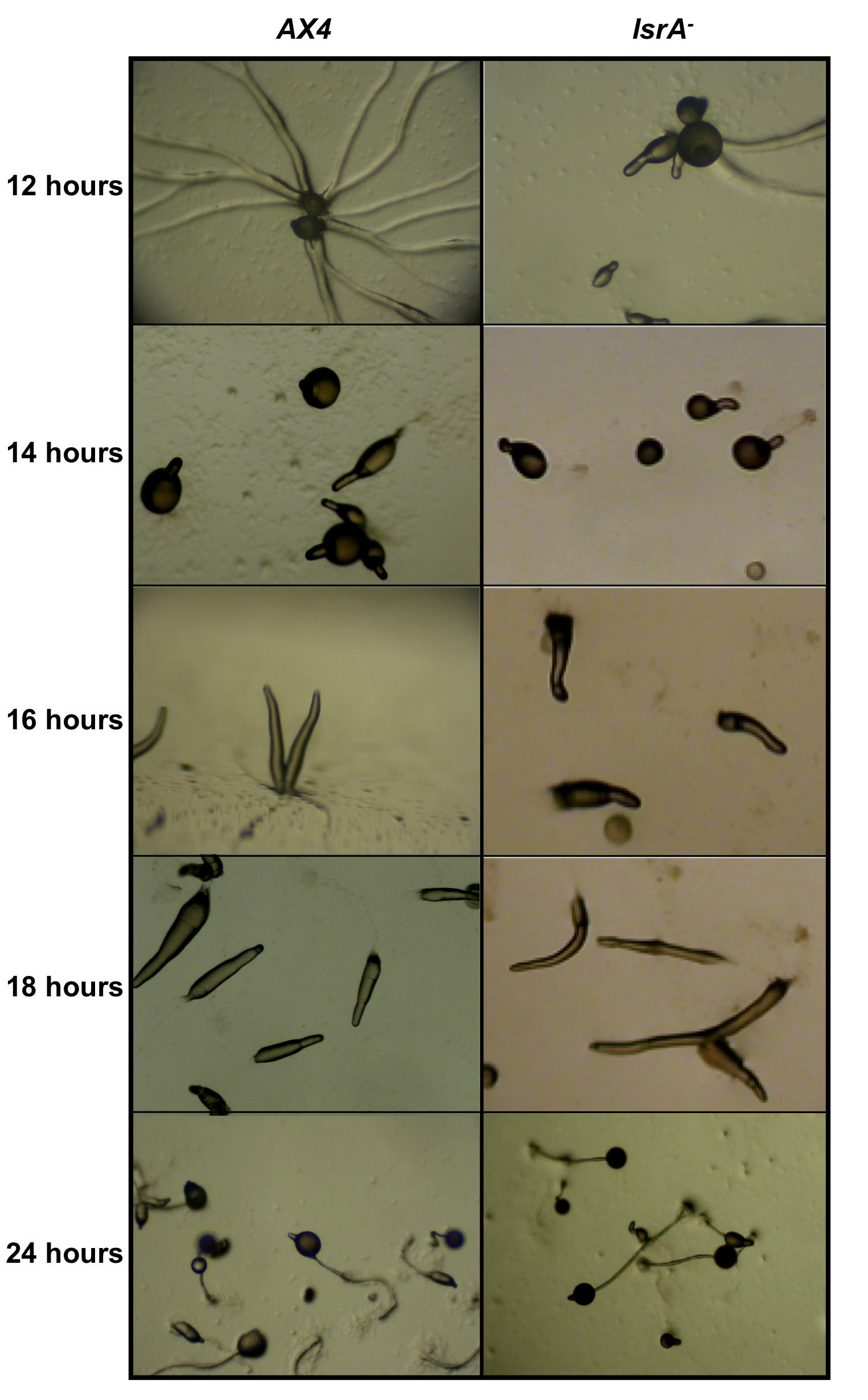

Supplement: Figure S1 — lsrA − does not exhibit obvious defects in developmental morphology or timing. lsrA − mutant and wild type cells were developed on non-nutrient agar for the times indicated. Both strains had reached equivalent stages at each time point. (TIF) [file pbio.1001039.s001.tif]

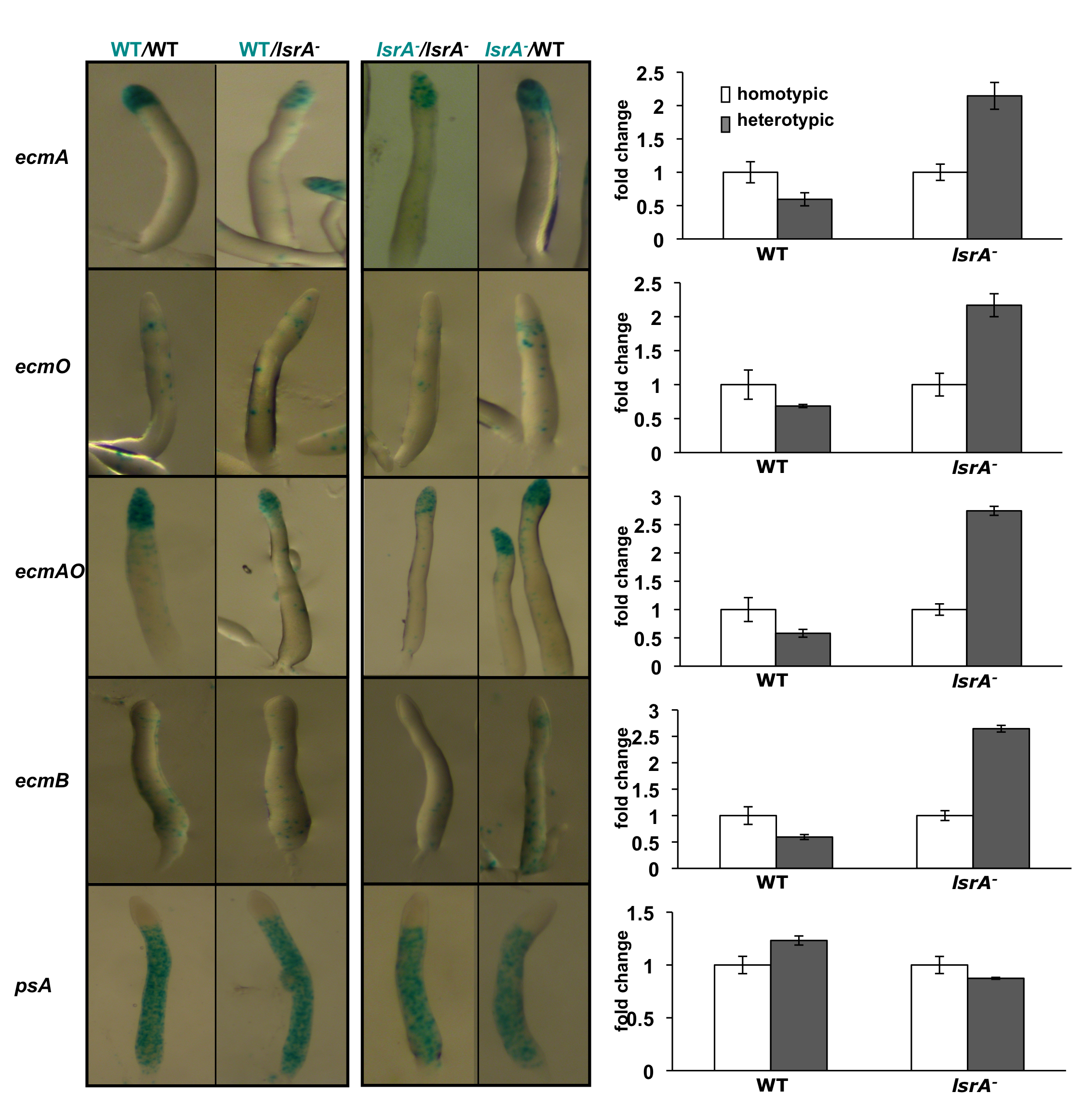

Supplement: Figure S2 — lsrA − exhibits general defects in prestalk cell differentiation when developed in chimera at slug stage. To test which prestalk cell types were affected in the lsrA − mutant, wild type and lsrA − mutant cells were transformed with lacZ markers that drive expression in each of the major prestalk (ecmA, ecmO, ecmAO, and ecmB) and prespore (psA) cell types. Strains expressing cell type–specific markers were mixed in chimera in a 10:90 ratio with unlabelled cells and relative expression assessed qualitatively and quantitatively at the slug stage. Wild type or lsrA − cells were transformed with cell-specific reporter genes. Clear differences were found in the expression of all prestalk cell-specific markers (ecmA, ecmO, ecmAO, and ecmB), although prespore cell-specific markers (psA) appear to be less affected. The expression of wild type prestalk markers was lower when mixed with a majority of mutant cells compared to when mixed with a majority of wild type cells. In contrast, the expression of mutant prestalk markers was higher when mixed with a majority of wild type cells compared to when mixed with a majority of mutant cells. To quantify this observation, the level of lacZ expression in heterotypic slugs was normalized to lacZ expression during homotypic development. The expression of wild type prestalk cell markers decreased when in chimera with mutant cells, whereas the expression of mutant prestalk cell markers increased when in chimera with wild type cells. The expression of the prespore marker showed the opposite pattern. The expression of wild type prespore marker increased when in chimera with mutant cells, whereas the expression of mutant prespore marker decreased when in chimera with wild type cells. Results are averages and standard deviations of three biological replicates, where each replicate was performed in triplicate. (TIF) [file pbio.1001039.s002.tif]

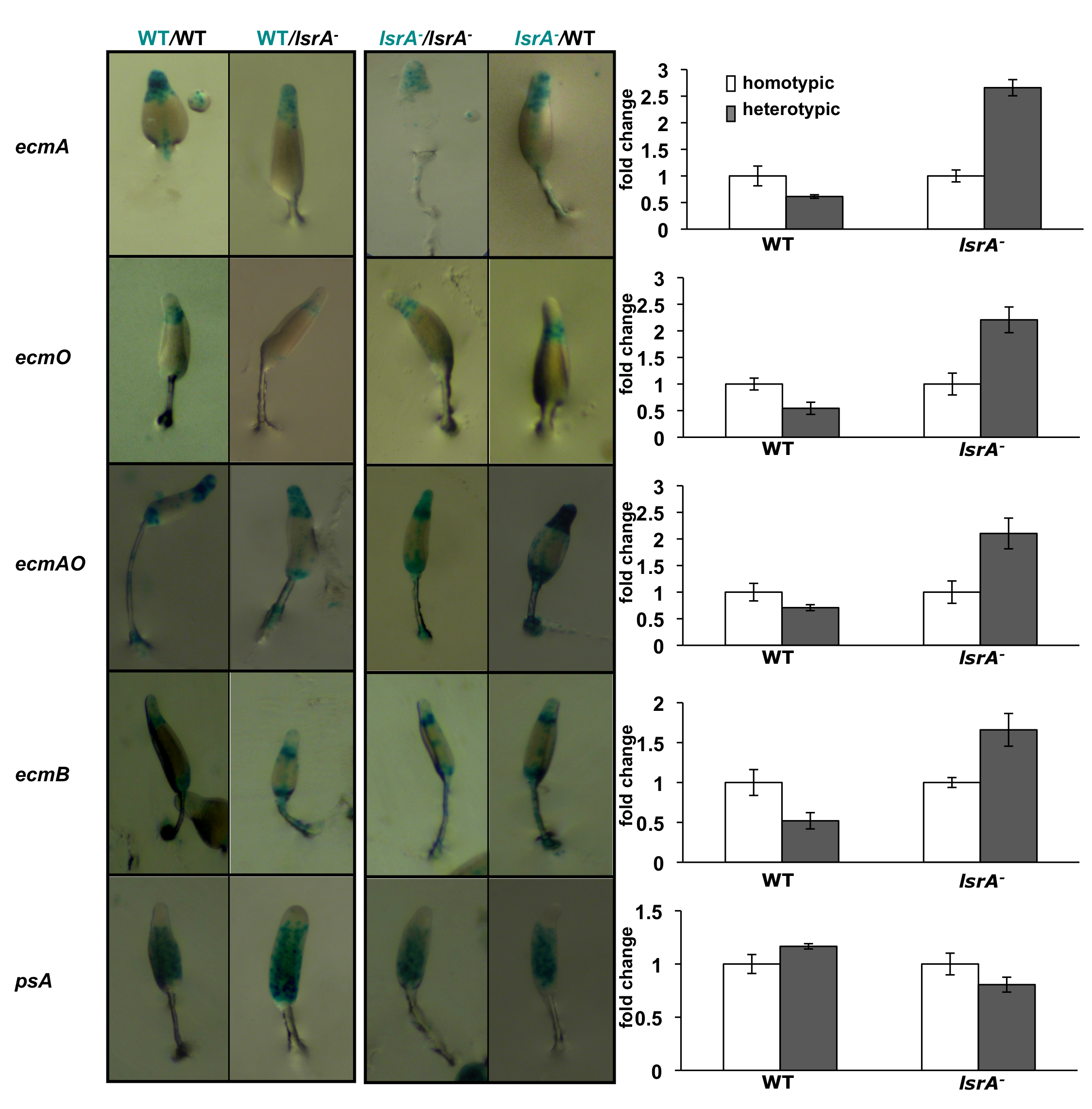

Supplement: Figure S3 — lsrA − exhibits general defects in prestalk cell differentiation when developed in chimera at culminant stage. To test which prestalk cell types were affected in the lsrA − mutant, wild type and lsrA − mutant cells were transformed with lacZ markers that drive expression in each of the major prestalk (ecmA, ecmO, ecmAO, and ecmB) and prespore (psA) cell types. Strains expressing cell type–specific markers were mixed in chimera in a 10:90 ratio with unlabelled cells and relative expression assessed qualitatively and quantitatively at the culminant stage. Wild type or lsrA − cells were transformed with cell-specific reporter genes. Clear differences were found in the expression of all prestalk cell-specific markers (ecmA, ecmO, ecmAO, and ecmB), although prespore cell-specific markers (psA) appear to be less affected. The expression of wild type prestalk markers was lower when mixed with a majority of mutant cells compared to when mixed with a majority of wild type cells. In contrast, the expression of mutant prestalk markers was higher when mixed with a majority of wild type cells compared to when mixed with a majority of mutant cells. To quantify this observation, the level of lacZ expression in heterotypic slugs was normalized to lacZ expression during homotypic development. The expression of wild type prestalk cell markers decreased when in chimera with mutant cells, whereas the expression of mutant prestalk cell markers increased when in chimera with wild type cells. The expression of the prespore marker showed the opposite pattern. The expression of wild type prespore marker increased when in chimera with mutant cells, whereas the expression of mutant prespore marker decreased when in chimera with wild type cells. Results are averages and standard deviations of three biological replicates, where each replicate was performed in triplicate. (TIF) [file pbio.1001039.s003.tif]

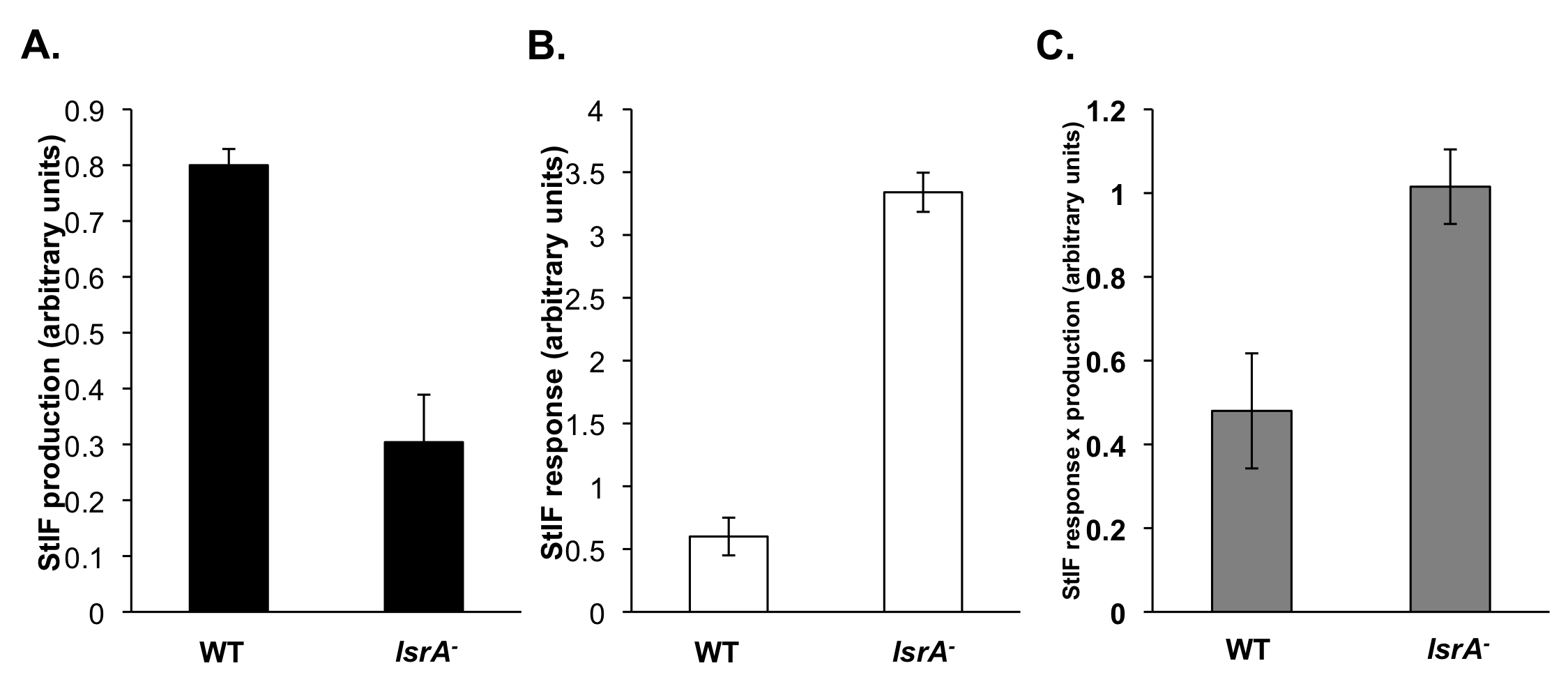

Supplement: Figure S4 — lsrA − cells exhibit differences in the responses to—and production of—StIFs. (A) Induction of ecmB-lacZ in wild type and lsrA − cells by StIF. Cells expressing ecmB-lacZ were developed in monolayer and gene expression induced by StIF. The response of lsrA − cells was 5.5-fold higher compared to wild type cells (t test, t 4 = 14.625, p<0.001). (B) Induction of ecmB-lacZ by StIFs collected from wild type and lsrA − cells. Cells expressing ecmB-lacZ were developed in monolayer and gene expression induced by StIFs collected from strains as indicated. Induction by lsrA − StIF was 0.38 times less compared to wild type StIF (t test, t 4 = 20.372, p<0.001). (C) Multiplying the response measurement by the production measurement can predict that the clonal stalk allocation of the lsrA − mutant is 2.12 times greater than wild type. (TIF) [file pbio.1001039.s004.tif]

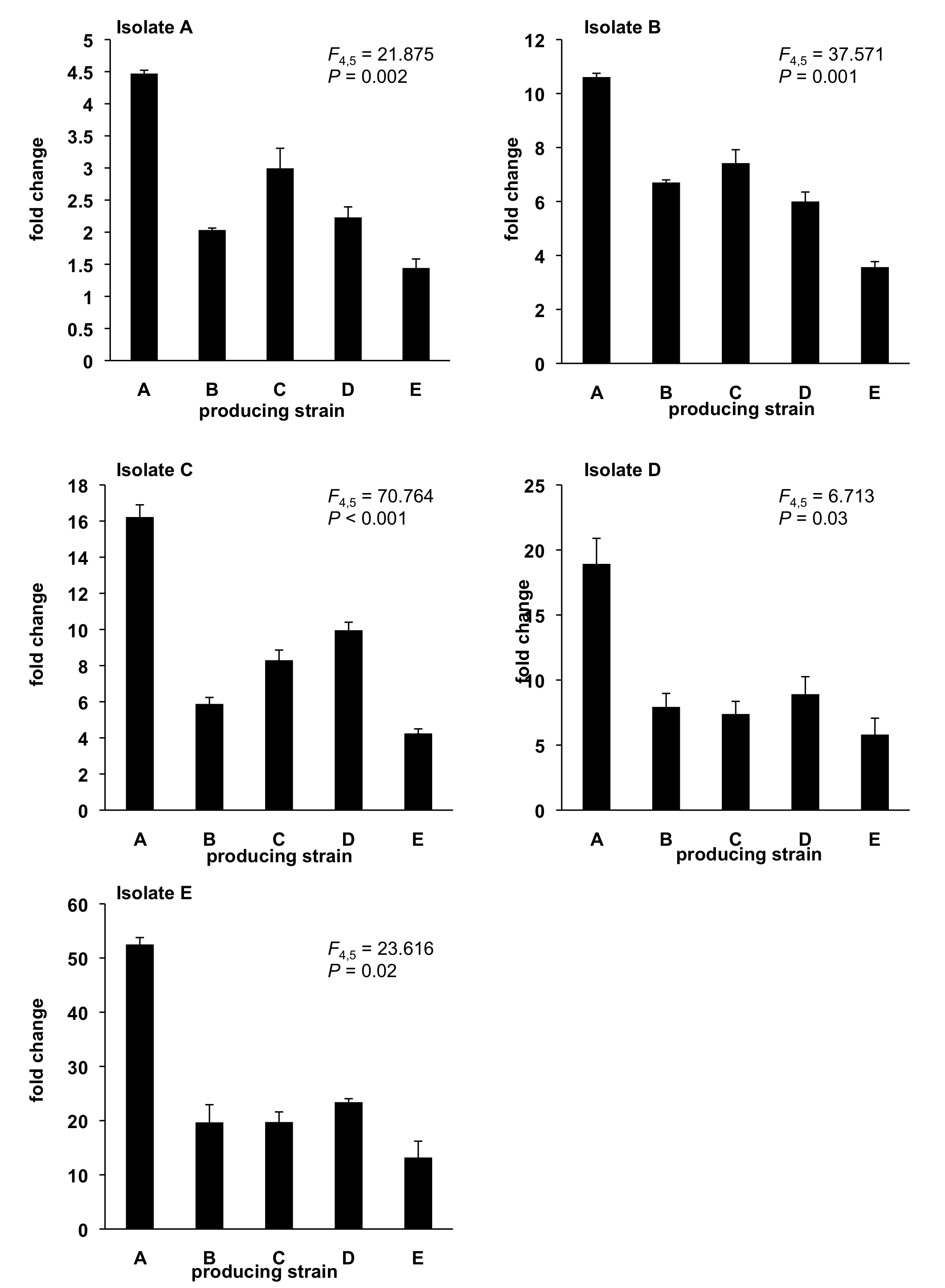

Supplement: Figure S5 — Natural isolates exhibit differences in the production of StIF. Induction of ecmAO-lacZ in natural isolates by StIF collected from each natural isolate. Cells of one isolate (indicated in the upper left-hand corner of each graph) expressing ecmAO-lacZ were developed in monolayer and gene expression measured in response to StIF collected from each isolate. Natural isolates vary dramatically in their production. Data are expressed as fold change in expression compared to no StIF control and are the average of three biological replicates. Significant differences in induction between strains were tested for using one-way ANOVAs. (TIF) [file pbio.1001039.s005.tif]

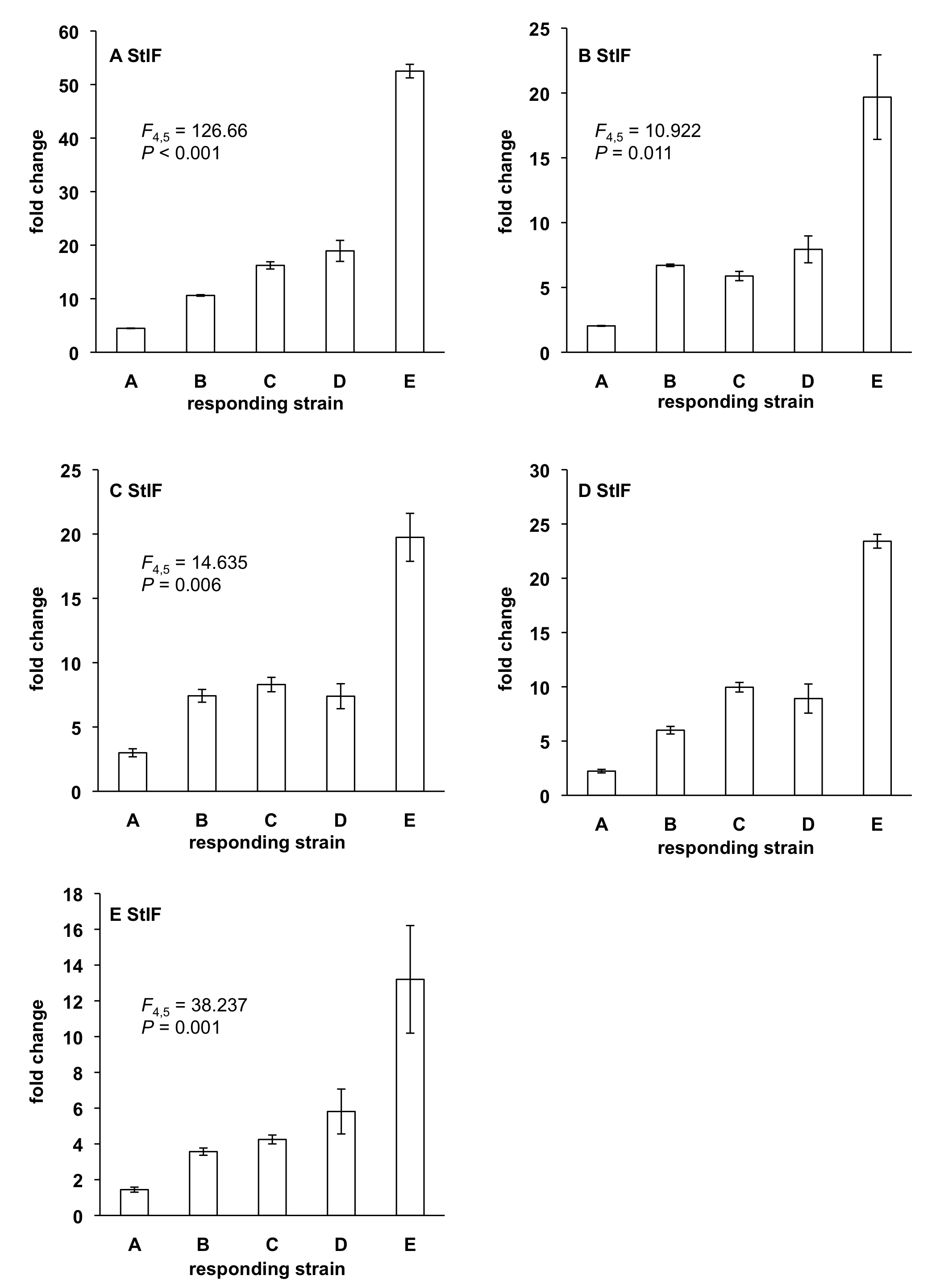

Supplement: Figure S6 — Natural isolates exhibit differences in the responses to StIFs. Induction of ecmAO-lacZ in natural isolates by StIF collected from each natural isolate (indicated in the upper left-hand corner of each graph). Different isolates expressing ecmAO-lacZ were developed in monolayer and gene expression measured in response to StIF from a single isolate. Natural isolates vary dramatically in their responsiveness, however the relative responses to each StIF from each isolate are comparable. Data are expressed as fold change in expression compared to no StIF control and are the average of three biological replicates. Significant differences in induction between strains were tested for using one-way ANOVAs. (TIF) [file pbio.1001039.s006.tif]
